# The effects of light therapy on depression and sleep in women during pregnancy or the postpartum period: A systematic review and meta‐analysis

**DOI:** 10.1002/brb3.3339

**Published:** 2023-11-29

**Authors:** Xinyu Li, Liang Fang, Lianzi Guan, Jiajia Zhang, Mingming Zheng, Daomin Zhu

**Affiliations:** ^1^ The School of Mental Health and Psychological Sciences Anhui Medical University Hefei Anhui China; ^2^ Department of Sleep Disorders Affiliated Psychological Hospital of Anhui Medical University Hefei Anhui China; ^3^ Department of Sleep Disorders Anhui Mental Health Center Hefei Anhui China; ^4^ Department of Sleep Disorders Hefei Fourth People's Hospital Hefei Anhui China

**Keywords:** depression, light therapy, pregnancy, sleep

## Abstract

**Background:**

In recent years, light therapy has been tried for the treatment of depression and sleep in pregnancy or postnatal period women, but the results have been inconclusive. This meta‐analysis is the first to systematically review the effects of light therapy on depression and sleep disturbances in women during pregnancy and the postnatal period.

**Methods:**

We searched for randomized controlled studies in PubMed, Embase, Cochrane Library, Web of Science, Chinese National Knowledge Infrastructure, and Chinese Biomedical Database up to January 2023. The standardized mean difference (SMD) was used to assess the efficacy of the outcome indicators.

**Results:**

Eight studies were eventually included in the analysis. The results showed that light therapy was more effective than the placebo group in terms of depression (SMD = .34, CI = .08–.61) and sleep (SMD = .64,95%CI = .28–1.00). Subgroup analysis could not explain the significant heterogeneity. There were no serious adverse effects in either the light therapy or placebo groups.

**Conclusions:**

Light therapy could be considered an effective treatment for depression and sleep disturbances in women during pregnancy and the postnatal period. However, future high‐quality trials with larger sample sizes are still needed.

## 1 INTRODUCTION

Women in the perinatal period suffer from the complication of depressive symptoms and sleep disturbances (Bei et al., 2015; Golenkov et al., 2019). According to statistics, the prevalence of depression during pregnancy and the postnatal period was 18.4% and 19.2% (Gavin et al., 2005a). On the other hand, pregnant women have more sleep disturbances (e.g., insomnia, insufficient sleep time, and poor sleep quality) than women who are not pregnant (Solomonova et al., 2020; Wilkerson & Uhde, 2018). These problems may result from a combination of hormonal, physical, and lifestyle changes occurring during the perinatal period. Women with these symptoms are likely to have difficulty building mother–child relationships, reduced intimacy, and, in severe cases, even harm themselves or their children, causing serious consequences for their families and individuals (Gavin et al., 2005b; Moehler et al., 2006). Currently, the main treatment for this type of disease is pharmacological treatments (Chaudhry & Susser, 2018). However, pharmacological treatments are severely teratogenic for infants and also have a negative impact on long‐term infant development (Byatt et al., 2013; Kallen & Otterblad, 2007; Reis & Kallen, 2010). Therefore, a safer treatment is needed to increase acceptance by pregnancy or postnatal period women.

Light therapy is performed by exposure for a period of time to a device that emits a certain intensity of light. Most common light therapies today require the patient to sit in front of an LED light box that emits a certain light intensity for a certain period of time. However, the optimal parameters for light therapy are currently inconclusive. As a safe, low‐cost physical therapy, it is now assumed that light therapy may function by stimulating intrinsically photosensitive retinal ganglion cells to improve depressed mood and sleep (Pail et al., 2011). Light therapy was first used to treat patients with the seasonal affective disorder (Lewy et al., 1982). The first open trial of light therapy on 16 pregnant women with major depressive disorder in 2002 showed significant improvements in depressive symptoms after the intervention (Oren et al., 2002). Subsequently, several studies have explored the efficacy of light therapy on depression and sleep in pregnancy or postnatal period women (Corral et al., 2007; Epperson et al., 2004). However, the sample sizes of these studies were small, the findings were not entirely consistent, and further investigation is needed to determine whether light therapy is effective for women in the pregnancy or postnatal period (Bais et al., 2021).

To our knowledge, this meta‐analysis is the first systematic review of the efficacy of light therapy on depression and sleep in women during pregnancy and the postpartum period. In this review, we not only explored the improvements in the light therapy depression and sleep scales before and after the intervention but also considered the effects of different subject characteristics, and interventions on efficacy.

## 2 METHODS

### 2.1 Search strategy

We conducted a systematic search for relevant studies in six databases, including PubMed, Embase, Cochrane Library, Web of Science, Chinese National Knowledge Infrastructure, and Chinese Biomedical Database. There were no restrictions on the publication date or language of the articles. The articles were searched up to January 2023. The keywords chosen for this search were “phototherapy,” “Light Therapy,” “Photoradiation Therapies,” “pregnancy,” “Mothers,” “Postpartum Period,” “Gestation,” “Postpartum,” and “Puerperium.” An example of the full electronic search strategy can be found in Supplementary S1. In addition, the reference lists of relevant articles included in the meta‐analysis were searched to avoid omissions.

### 2.2 Inclusion and exclusion criteria

This systematic review of previous systematic reviews with meta‐analysis is registered in the International Prospective Register of Systematic Reviews (PROSPERO) trial registry (CRD42023391474). In addition, and where applicable, the general guidelines of the Preferred Reporting Items for Systematic Reviews and Meta‐Analysis (PRISMA) statement were followed (Liberati et al., 2009).

Studies meeting the following criteria were considered for inclusion in the meta‐analysis: (1) Subjects were women in pregnancy or the first year postpartum; (2) studies were limited to human studies; (3) randomized controlled studies (RCTs), including intervention and control groups; (4) light therapy was used as the primary intervention, with no restrictions on the equipment, intensity, duration of treatment, or time of light; (5) the control group was usually dim red light, low‐intensity light, or no treatment; (6) standardized and validated depression or sleep assessment scales were used in the study, such as the Edinburgh Postnatal Depression Scale (EPDS) (Cox et al., 1987), the Hamilton Depression Scale‐Seasonal Affective Disorder Version (SIGH‐SAD) (Williams, 1988), the Pittsburgh Sleep Quality Index (PSQI), or the Insomnia Severity Index (ISI). Exclusion criteria were as follows: (1) exclusion of reviews, meta‐analyses, and case reports; (2) studies that combined other treatment options, such as light therapy combined with cognitive behavioral therapy, to assess the effectiveness of adjunctive light therapy itself.

### 2.3 Data extraction

**FIGURE 1:**
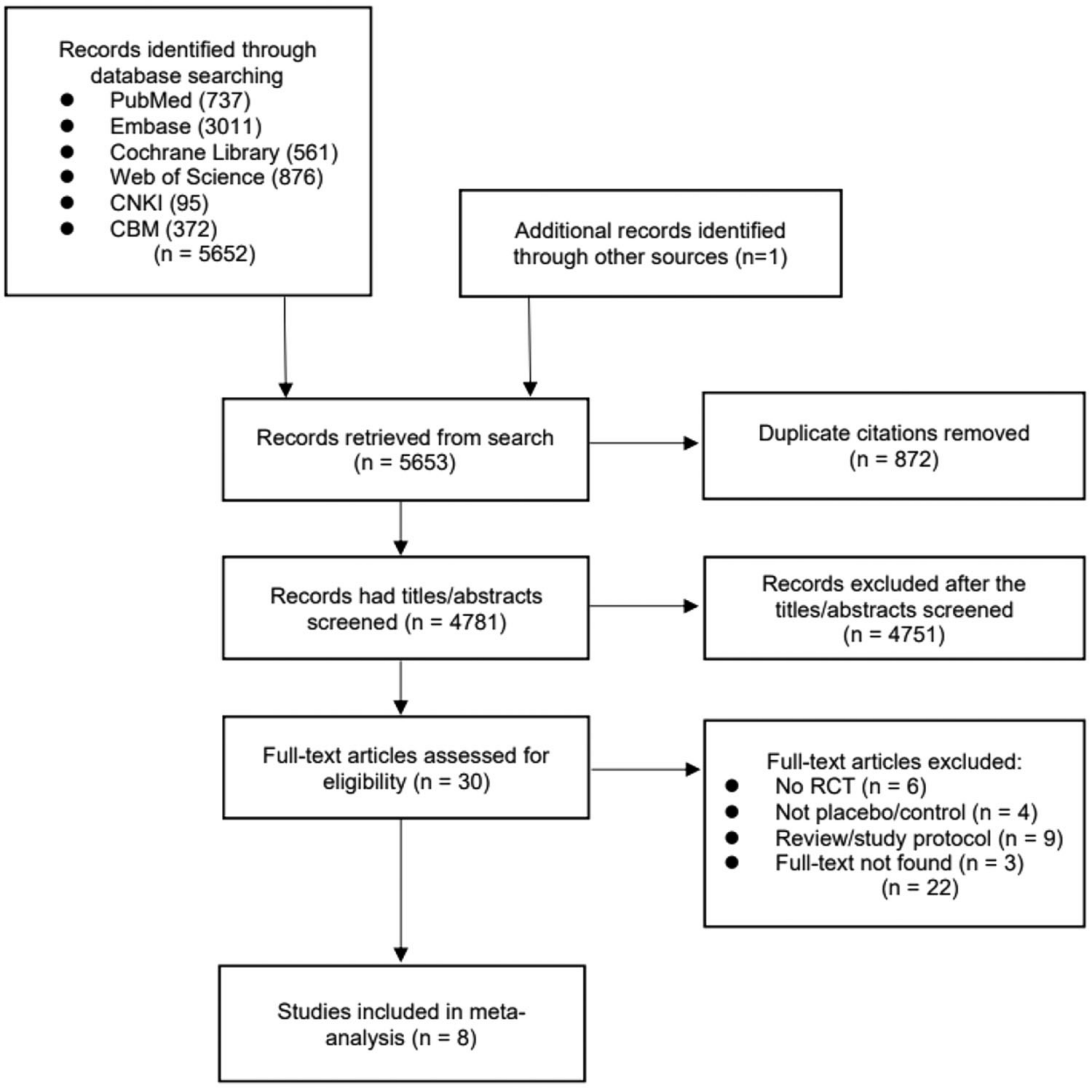
Flow diagram of the study selection process.

All steps of the screening and data extraction for the study were performed by two independent reviewers (Li and Fang). The names of the first author, year of publication, study design, sample size, subject characteristics, details of the intervention and control groups, and outcome indicators were extracted for the studies. In cases of inconsistency, a third reviewer was involved in the discussion to reach a consensus. See Figure 1 for a flow diagram of the study.

### 2.4 Assessment of risk of bias and quality of evidence

The risk of bias was evaluated using the Cochrane Collaboration's “Risk of Bias 2.0” tool (Higgins et al., 2011) in seven domains: random sequence generation, allocation concealment, blinding of participants and personnel, blinding of the outcome assessment, incomplete outcome data, selective reporting, and other bias, using “low bias,” “unclear,” or “high bias” for each indicator. The overall quality of evidence was examined using the Grading of Recommendations Assessment, Development and Evaluation (GRADE) system (version 3.6) (Guyatt et al., 2011).

### 2.5 Statistical analysis

The meta‐analysis was conducted using Cochrane Collaboration's Review Manager software (version 5.3.0). We compared mean differences from baseline to endpoint for each group. In addition, the included trials used different measurement scales to quantify the degree of depression and sleep disturbance, and therefore, standardized mean differences (SMDs) were used to compare results using the same metric. Heterogeneity among included studies was assessed using the *Q*‐test and the *I* ^2^ statistic. *I* ^2^ values of 25% were classified as low heterogeneity, 50% as moderate heterogeneity, and 75% as high heterogeneity (Ioannidis, 2008). We chose a random effects model for significant heterogeneity (*I* ^2^ > 50%) (Borenstein et al., 2010a). Otherwise, a fixed effects model (*I* ^2^ ≤ 50%) was used (Borenstein et al., 2010b). Funnel plots were used to assess for potential publication bias. In addition, sensitivity analyses were conducted using a leave‐one‐out approach, removing each study individually and analyzing the overall impact on the pooled estimates and/or heterogeneity (Higgins, 2008).

### 2.6 Subgroup analyses

1. Subgroup analysis was performed according to whether the subjects were pregnant or in the postpartum period. This is because we did not find conclusive evidence of differences in the efficacy of light therapy for women at different periods.
2. Little comparison exists for the use of light therapy in the two settings, outpatient and inpatient (Oldham & Ciraulo, 2014). Because outpatient subjects may have fewer depressive symptoms and insomnia on average, a subgroup analysis of inpatient or outpatient was performed as a proxy for the severity of depression or sleep disturbance.
3. We conducted a subgroup analysis of the time of light therapy (<30 min or >30 min). We hypothesized that longer periods of light would have a greater effect on depression and sleep improvement.
4. Subgroup analysis was performed for light therapy duration of fewer than 6 weeks or more than 6 weeks. We hypothesize that a longer duration of light therapy is more effective (Al‐Karawi & Jubair, 2016).

## 3 RESULTS

### 3.1 Study characteristics

After searching the database, we found 5652 documents and 1 through other sources. After excluding 872 duplicates, we screened the titles, abstracts, and full texts progressively. In the final, 8 studies, including 258 subjects, were included in this meta‐analysis. Details of each study are shown in Table 1.

**TABLE 1:** Study characteristics.

Study details					Light therapy details and adjuncts			Intervention information		Control information	
Study (author, year)	Depression scale	Sleep scale	Prenatal/Postnatal	Inpatient/Outpatient	Duration of treatment	Intervention	Control	Number	Age: mean (SD)	Number	Age: mean (SD)
Donmez et al. (2022)	EPDS	PSQI	Postnatal	Outpatient	3 weeks	10,000 lx, 45 min/day, after awakening (preferably before 10 a.m.)	500 lx, 45 min/day, after awakening (preferably before 10 a.m.)	*n* = 12	29.73(6.6)	*n* = 11	28(3.8)
Garbazza et al. (2022)	EPDS		Postnatal	Outpatient	6 weeks	10,000 lx bright light, 30 min/day, within 20 min after awakening	19 lx dim red light, 30 min/day, within 20 min after awakening	*n* = 11	median age:33	*n* = 11	median age:32
Verma et al. (2022)	PROMIS‐ Depression	ISI	Postnatal	Outpatient	6 weeks	1250 lx blue‐enriched white light, 20 min/day, within 20 min after awakening	Treatment as usual	*n* = 36	32.35(5.0)	*n* = 39	31.42(3.77)
Bais et al. (2020)	EPDS		Prenatal	Inpatient	6 weeks	9000 lx bright light, 30 min/day, within 30 min after awakening	100 lx dim light, 30 min/day, within 30 min after awakening	*n* = 28	31.9(4.4)	*n* = 28	31.9(5.3)
Lee et al. (2013)	EPDS	GSDS	Postnatal	Inpatient	3 weeks	8000 lx blue–green light, 30 min/day, within 60 min after awakening	5 lx red light, 30 min/day, within 60 min after awakening	*n* = 16	24.4(5.4)	*n* = 14	29.1(6.7)
Wirz‐Justice et al. (2011)	SIGH‐ADS HDRS		Prenatal	Outpatient	5 weeks	7000 lx white light, 60 min/day, within 10 min after awakening	70 lx red light, 60 min/day, within 10 min after awakening	*n* = 16	31.7(4.7)	*n* = 11	32.7(5.4)
Corral et al. (2007)	EPDS		Postnatal	Outpatient	4 weeks	10,000 lx bright light, 30 min/day, within 20 min after awakening	600 lx red light, 30 min/day, within 20 min after awakening	*n* = 10	34.6(4.0)	*n* = 5	33.6(2.1)
Epperson et al. (2004)	SIGH‐SAD		Prenatal	Outpatient	5 weeks	7000 lx bright light, 60 min/day, within 10 min after awakening	500 lx dim light, 60 min/day, within 10 min after awakening	*n* = 5	32.2(5.4)	*n* = 5	34(1.4)

Abbreviations: CSD, consensus sleep diary; EPDS, Edinburgh Postnatal Depression Scale; ESS, Epworth sleepiness scale; GSDS, general sleep disturbance scale; HAMD, Hamilton Depression Scale; ISI, insomnia severity index; MADRS, Montgomery–Åsberg Depression Rating Scale; MEQ, morningness–eveningness questionnaire; PROMIS Depression, PROMIS Depression‐Short Form‐8a; PSQI, Pittsburgh sleep quality index; SIGH‐ADS, Structured Interview Guide for the Hamilton Depression Rating Scale‐Atypical Depression Supplement; SIGH‐SAD, structured interview guide for the Hamilton Depression Rating Scale‐Seasonal Affective Disorder; SPAQ, seasonal pattern assessment questionnaire.

The average age of participants in each study ranged from 24 to 34 years. The light equipment for the two trials were light glasses and light visors, respectively (Lee et al., 2013; Verma et al., 2022), and the rest used light boxes. The intensity of light therapy ranges from 1250 to 10,000 lx, and all studies had morning treatment sessions. The daily treatment duration ranged from 30 to 60 min, and the duration of treatment varied between 3 and 6 weeks. All trial controls were <1000 lx dim light, except for one study that used the usual treatment as a control (Verma et al., 2022).

For depression scales, four studies used the EPDS, one study used the SIGH‐SAD, one study used the Hamilton Depression Rating Scale (HAMILTON, 1960), and one employed the PROMIS Depression‐Short Form‐8a (PROMIS Depression) (Pilkonis et al., 2011). The sleep scales used the PSQI (Buysse et al., 1989), ISI (Bastien et al., 2001), and sleep quality in the General Sleep Disturbance scale (Lee, 1992).

### 3.2 Effect of light therapy on depression

**FIGURE 2:**
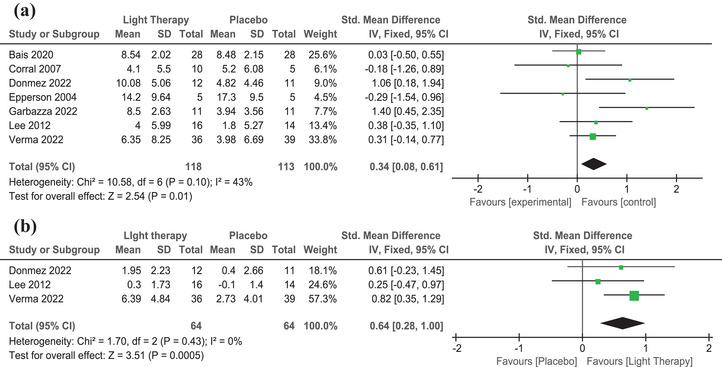
Forest plots of SMD in light therapy (a. Depression, b. sleep).

We included the final eight studies in the meta‐analysis and found a high degree of heterogeneity (*I* ^2^ = 75%). After excluding one study using the leave‐one‐out method (Wirz‐Justice et al., 2011), heterogeneity was reduced to *I* ^2^ = 43% and had no significant effect on the results. The SMD for improvement in depression scores before and after the intervention was calculated for a total of 231 subjects from the 7 included studies. The results of the meta‐analysis forest plot showed that depressive symptoms improved better in the intervention group than in the control group after receiving light therapy (SMD = .34, CI = .08–.61; *z* = 2.54, *p* = .01) (Figure 2a).

### 3.3 Effect of light therapy on sleep

In this analysis, only 3 of the 8 studies (128 subjects) provided data for the sleep assessment (Figure 2b). There was a significant difference between the improvement in sleep scores of the intervention group and the control group (SMD = .64,95%CI = .28–1.00; *z* = 3.51, *p* < .001). This suggests that light therapy could improve sleep in women during pregnancy or the postpartum period.

### 3.4 The result of the subgroup analysis

The results of the first subgroup analysis showed the different effects on depression improvement induced by light therapy for women in the prenatal and postnatal periods. As shown in Table 2, the prenatal group showed significant heterogeneity (*I* ^2^ = 90%, *p* < .01). The heterogeneity in the postnatal group was moderate (*I* ^2^ = 45%, *p* = .02). The effect of light therapy was significant in the postnatal group (SMD = .5, 95% CI = .18–.82, *p* = .002). However, it was not significant in the prenatal group (SMD = .43, 95% CI = −.02 to .87, *p* > .05).

**TABLE 2:** Subgroup analysis of the efficacy of light therapy on depressive symptoms.

Subgroups	No	*I* ^2^ (%)	SMD (95%CI)	*p*
Prenatal/Postnatal				
Prenatal	3	90	.43[−.02,.87]	NS
Postnatal	5	45	.5[.18,.82]	.002
Inpatient/Outpatient				
Inpatient	2	0	.15[−.28,.57]	NS
Outpatient	5	79	.66[.34,.99]	<.001
Time				
Short‐time (≤30 min)	4	57	.29[−.02,.60]	NS
Long‐time (> 30 min)	4	82	.89[.43,1.36]	<.001
Duration of treatment				
Short‐duration(< 6 weeks)	4	84	.91[.38,1.43]	<.001
Long‐duration (≥6 weeks)	4	51	.34[.04,.63]	<.001

Abbreviations: CI, confidence interval; NS, not significant; SMD, standardized mean difference.

To examine the effects of depression and sleep disorder severity, we chose a second subgroup analysis of the different effects induced by light therapy on inpatient and outpatient subjects. As shown in Table 2, there was significant heterogeneity in the outpatient group (*I* ^2^ = 79%, *p* < .001) with an SMD of .66 (95% CI = .34–.99, *p* < .001), whereas the inpatient group was not significantly heterogeneous (*I* ^2^ = 0%, *p* = .44) and had a non‐significant SMD of .15 (95% CI = −.28 to .57, *p* = .49). Inpatient and outpatient statuses could not be used to explain the observed heterogeneity.

Considering that the efficacy of light therapy may depend on the duration of treatment, we performed subgroup analyses for different treatment durations. Both the short‐time group (*I* ^2^ = 57%, *p* = .07) and the long‐time group (*I* ^2^ = 82%, *p* = .001) showed significant heterogeneity. Results (Table 2) consistent with our hypothesis indicated that there was a significant effect of light therapy for the long‐time group (SMD = .89, 95% CI = .43–1.36, *p* < .001) and a non‐significant effect for the short‐time group (SMD = .29, 95% CI = −.02 to .6, *p* > .05).

The last subgroup analysis showed that the light duration of more or less than 6 weeks could cause different effects. Table 2 shows significant heterogeneity between the short‐duration group (*I* ^2^ = 81%, *p* < .001) and the long‐duration group (*I* ^2^ = 51%, *p* = .10). Contrary to our hypothesis, the short‐duration group (SMD = .91, 95% CI = .83–1.43, *p* < .001) was more effective in reducing depressive symptoms than the long‐duration group (SMD = .34, 95% CI = .04–.63, *p* = .02).

### 3.5 Risk of bias assessment and publication bias

**FIGURE 3:**
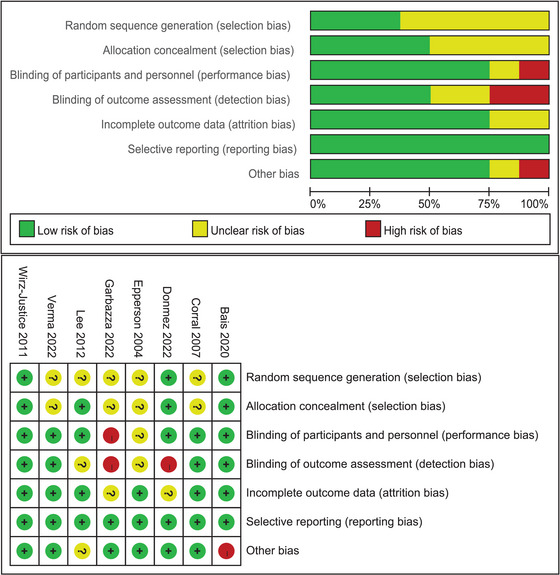
Risk of bias graph.

Figure 3 summarizes the risk of bias assessments using the Cochrane Risk of Bias Tool. Most studies were considered to have a “high” or “unclear” risk of bias in at least one of the basic methodological criteria. Random sequence generation (mainly due to under‐reporting and concealment of the generation of allocation sequences) and allocation concealment (mainly inadequate reporting of the researcher's knowledge of the allocation group) were the most common sources of bias. There appears to be a fair amount of symmetry in the shape of the funnel plot (Supplementary S2), suggesting little evidence of bias.

### 3.6 Sensitivity analysis

For the analysis of depression, we excluded one study from the sensitivity analysis using leave‐one‐out analysis (Wirz‐Justice et al., 2011), resulting in greatly reduced heterogeneity (see Supplementary S3 for forest plots before exclusion). There was no significant change in the final combined estimates of the seven included studies (SMD .25–.45). For sleep outcomes, there was again no significant change in the combined estimates of the three included studies only (SMD .4–.77).

### 3.7 Adverse events

A total of four of the eight included studies reported adverse effects associated with light therapy (Bais et al., 2020; Donmez et al., 2022; Verma et al., 2022; Wirz‐Justice et al., 2011), with no studies reporting the occurrence of serious adverse effects. For women treated with light, the most common adverse effect was headaches. A total of 18 participants reported headaches, and only 1 withdrew because of intolerable conditions (Donmez et al., 2022), and all resolved on their own after a few days. In addition to this, four participants reported sleep problems, three were nauseous and two were dizzy (Bais et al., 2020; Verma et al., 2022).

## 4 DISCUSSION

The current systematic review and meta‐analysis aims to investigate the efficacy of light therapy in the treatment of depression and sleep disturbances in women during pregnancy and the postpartum period. Following an extensive literature search, eight studies were included in the meta‐analysis. The results of this study showed that light therapy had a significant mild‐to‐moderate effect on the improvement of depressive symptoms in perinatal women and a moderately significant effect on sleep disturbances compared to placebo/control treatment. Subgroup analysis showed that light therapy was more effective for women in the postpartum period, when they received treatment in an outpatient setting, when the light therapy time was more than 30 min, and the duration of the treatment did not exceed 6 weeks. In addition, our findings in terms of adverse effects showed that there were no serious adverse effects in the light therapy group versus the control group. Mild adverse reactions, including headaches, sleep problems, nauseas, and dizziness, were well tolerated.

It has been suggested that pregnancy leads to changes in the hypothalamus–pituitary–adrenal axis (HPA axis) (Seth et al., 2016). At the end of pregnancy, the mother's cortisol levels maybe 60–700 times higher than before pregnancy and therefore may increase susceptibility to perinatal depression (Campbell et al., 1987). Light therapy may improve depressive symptoms in perinatal women by influencing their HPA axis. Our meta‐analysis further showed that light therapy was significantly effective in treating depressive symptoms in perinatal women. Our results showed mild‐to‐moderate efficacy for depression (SMD = .34), which is similar to the SMD reported by Pjrek et al. (2020) (SMD = .37) and Tao et al. (2020) (SMD = .40) for seasonal/non‐seasonal affective disorder. On the other hand, improvement in sleep may be through modulation of circadian rhythm phase or amplitude (Bais et al., 2021). Further studies are needed in the future to determine the possible mechanisms.

In addition, four subgroup analyses were performed. Interestingly, a light duration of no more than 6 weeks was shown to have better effects, which is inconsistent with our hypothesis. Martensson et al. (2015) reported similar findings in a meta‐analysis that light therapy had a significant effect only at 2–3 weeks of treatment. This might be related to the fact that spending too much time per day on treatment may reduce treatment adherence. This suggests that long‐duration light therapy may not increase the antidepressant effect and that short‐duration light therapy may be a better option for future treatment. More rigorous RCTs are needed in the future to explore the mechanisms by which the duration of light therapy affects efficacy.

This meta‐analysis found a significant effect of light therapy on sleep in perinatal women. This is consistent with the results of a meta‐analysis by van Maanen et al. (2016) on the effects of light therapy on sleep problems. Perinatal women often experience sleep disturbances, which may be due to various hormonal changes and disturbed sleep patterns (Sahota et al., 2003; Santiago et al., 2001). Light therapy acts as a powerful external timing factor, exerting its therapeutic effect by correcting abnormal melatonin phasing (Lewy et al., 1988; Tamura et al., 2008; Zisapel, 2018). However, only three RCTs were included in this meta‐analysis for sleep. More and larger sample sizes of studies are needed in the future to further explore the effects of light therapy on sleep in perinatal women.

It is worth considering that four people in the one study included in this trial reported adverse effects that resulted in sleep problems (Bais et al., 2020), which contradicts the conclusion of this study that light therapy improves sleep. However, this study did not assess subjects for baseline sleep problems, and three people in the light therapy group in this study were taking sleep medications, which means that some of the subjects had sleep problems themselves. Therefore, we were unable to assess changes in sleep in these subjects after light therapy.

The overall quality of the evidence was assessed by the GRADE system. We concluded that there is very little evidence to support the efficacy of light therapy for depression and sleep in women during pregnancy and the postpartum period (Supplementary S4). The biggest reason for this is the inconsistency between studies in the parameters of treatment and the scales used to assess outcomes.

There are several potential limitations of this study. First, moderate heterogeneity was present in the study. We did not find a source of heterogeneity that could be explained by factors based on all subgroup analyses. Second, the sample sizes of some of these studies were quite small, which may have explained some of the nonsignificant findings of depression in the single studies. Finally, the long‐term effects of light therapy were not explored as we only estimated the improvement in depression based on the mean change in depression scores after therapy and did not assess post‐intervention follow‐up.

## 5 CONCLUSION

This meta‐analysis shows that light therapy is effective for depression and sleep in women during pregnancy and the postpartum period. The subgroup analysis suggested that outpatient postpartum patients with a light therapy time of no less than 60 min/day, and a duration of fewer than 6 weeks may be more effective. In addition, this review showed that there were no serious side effects between the light therapy and placebo groups. However, the number of RCT design studies and sample sizes available for this review were limited, and therefore, more future studies on light therapy for depression and sleep in perinatal women are needed.

## AUTHOR CONTRIBUTIONS

**Xinyu Li**: Conceptualization; validation; investigation; data curation; writing—original draft; visualization. **Liang Fang**: Validation; methodology; software. **Lianzi Guan**: Software; validation; methodology. **Jiajia Zhang**: Writing—review and editing; supervision. **Mingming Zheng**: Supervision; writing—review and editing. **Daomin Zhu**: Writing—review and editing; supervision; funding acquisition.

## CONFLICT OF INTEREST STATEMENT

All authors declare that they have no conflicts of interest.

### PEER REVIEW

The peer review history for this article is available at https://publons.com/publon/10.1002/brb3.3339

## Supporting information

Supporting information

## Data Availability

Data sharing is not applicable to this article as no new data were created or analyzed in this study. The data that support the findings are available in the manuscript.
