# Supplementary material for: The effects of light therapy on depression and sleep in women during pregnancy or the postpartum period: A systematic review and meta‐analysis
Source: Brain Behav. 2023 Nov 29;13(12):e3339. doi: 10.1002/brb3.3339 (PMC10726786; doi:10.1002/brb3.3339)
Supplement: Supplementary file 1 — Supporting information [file BRB3-13-e3339-s001.docx]

Supplementary

**S1. Search Strategy**

| Database | Search Strategy | |
| --- | --- | --- |
| Pubmed | #1 | ("Phototherapy"[Mesh]) OR ((((((((((Phototherapies[Title/Abstract]) OR (Therapy, Photoradiation[Title/Abstract])) OR (Photoradiation Therapies[Title/Abstract])) OR (Therapies, Photoradiation[Title/Abstract])) OR (Light Therapy[Title/Abstract])) OR (Light Therapies[Title/Abstract])) OR (Therapies, Light[Title/Abstract])) OR (Therapy, Light[Title/Abstract])) OR (Photoradiation Therapy[Title/Abstract])) |
|  | #2 | ((("Pregnancy"[Mesh]) OR ("Mothers"[Mesh])) OR ("Postpartum Period"[Mesh])) OR ((((((((Pregnancies[Title/Abstract]) OR (Gestation[Title/Abstract])) OR (Mother[Title/Abstract])) OR (Period, Postpartum[Title/Abstract])) OR (Postpartum[Title/Abstract])) OR (Postpartum Women[Title/Abstract])) OR (Women, Postpartum[Title/Abstract])) OR (Puerperium[Title/Abstract])) |
|  | #3 | #1 AND #2 |
| Cochrane Library | #1 | MeSH descriptor：[Phototherapy] explode all trees |
|  | #2 | Phototherapies |
|  | #3 | Therapy, Photoradiation |
|  | #4 | Photoradiation Therapies |
|  | #5 | Therapies, Photoradiation |
|  | #6 | Light Therapy |
|  | #7 | Light Therapies |
|  | #8 | Therapies, Light |
|  | #9 | Therapy, Light |
|  | #10 | Photoradiation Therapy |
|  | #11 | #1 OR #2 OR #3 OR #4 OR #5 OR #6 OR #7 OR #8 OR #9 OR #10 |
|  | #12 | MeSH descriptor: [Pregnancy] explode all trees |
|  | #13 | MeSH descriptor: [Mothers] explode all trees |
|  | #14 | MeSH descriptor: [Postpartum Period] explode all trees |
|  | #15 | Pregnancies |
|  | #16 | Gestation |
|  | #17 | Mother |
|  | #18 | Period, Postpartum |
|  | #19 | Postpartum |
|  | #20 | Postpartum Women |
|  | #21 | Women, Postpartum |
|  | #22 | Puerperium |
|  | #23 | #12 OR #13 OR #14 OR #15 OR #16 OR #17 OR #18 OR #19 OR #20 OR #21 OR #22 |
|  | #24 | #11 AND #23 |
| Embase | #1 | phototherapy'/exp |
|  | #2 | Phototherapies:ti,ab,kw OR 'Therapy, Photoradiation':ti,ab,kw OR 'Photoradiation Therapies':ti,ab,kw OR 'Therapies, Photoradiation':ti,ab,kw OR 'Light Therapy':ti,ab,kw OR 'Light Therapies':ti,ab,kw OR 'Therapies, Light':ti,ab,kw OR 'Therapy, Light':ti,ab,kw OR 'Photoradiation Therapy':ti,ab,kw |
|  | #3 | #1 OR #2 |
|  | #4 | 'puerperium'/exp |
|  | #5 | 'mothers'/exp |
|  | #6 | 'pregnancy'/exp |
|  | #7 | 'postpartum period':ti,ab,kw OR 'pregnancies':ti,ab,kw OR 'gestation':ti,ab,kw OR 'mother':ti,ab,kw OR 'period, postpartum':ti,ab,kw OR 'postpartum':ti,ab,kw OR 'postpartum women':ti,ab,kw OR 'women, postpartum':ti,ab,kw |
|  | #8 | #4 OR #5 OR #6 OR #7 |
|  | #9 | #3 AND #8 |
| Web of Science Core Collection | #1 | TS=(phototherapy OR Phototherapies OR "Therapy, Photoradiation" OR "Photoradiation Therapies" OR "Therapies, Photoradiation" OR "Light Therapy" OR "Light Therapies" OR "Therapies, Light" OR "Therapy, Light" OR "Photoradiation Therapy" ) |
|  | #2 | TS=(Pregnancy OR Mothers OR "Postpartum Period" OR Pregnancies OR Gestation OR Mother OR "Period, Postpartum" OR Postpartum OR "Postpartum Women" OR "Women, Postpartum" OR Puerperium) |
|  | #3 | #1 AND #2 |
| Chinese National Knowledge Infrastructure（CNKI） | #1 | SU %= '光疗法' OR TKA = '光照治疗'+'光疗'+'强光治疗'+'强光疗法' |
|  | #2 | SU %= '围产期' OR SU %= '产后期' OR SU %= '孕妇' OR SU %= '母亲' OR SU %= '妊娠' OR TKA = '围生期'+'妊娠期' |
|  | #3 | #1 AND #2 |
| Chinese Biomedical Database(CBM) | #1 | ("光疗法"[加权:扩展]) OR ("光照治疗"[常用字段:智能] OR "光疗"[常用字段:智能] OR "强光疗法"[常用字段:智能] OR "强光治疗"[常用字段:智能]) |
|  | #2 | ("围产期"[加权:扩展]) OR ("孕妇"[加权:扩展]) OR ("产后期"[加权:扩展]) OR ("母亲"[加权:扩展]) OR ("围生期"[常用字段:智能] OR "妊娠"[常用字段:智能]) |
|  | #3 | #1 AND #2 |

**S2. Funnel plot**

**
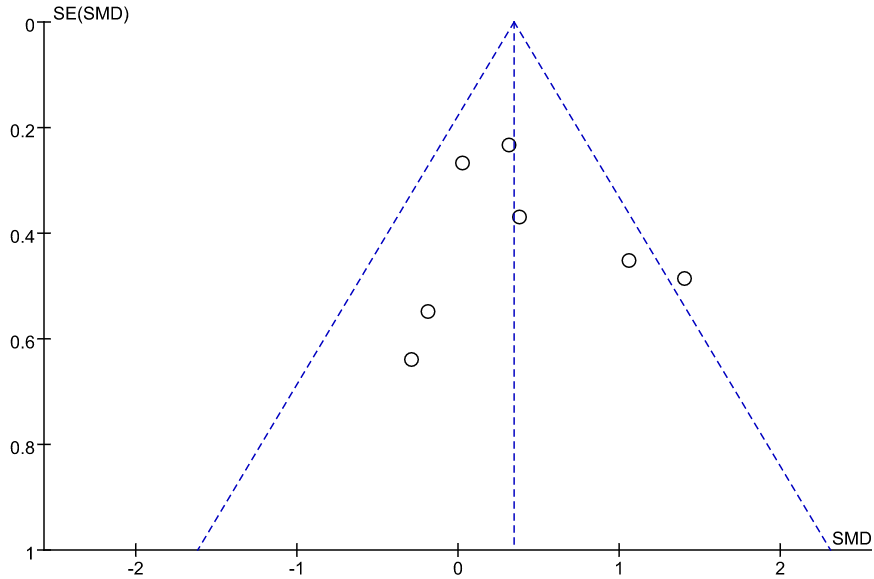
**

**S3. Forest plot of the effects of light therapy for depression (before excluding)**

**
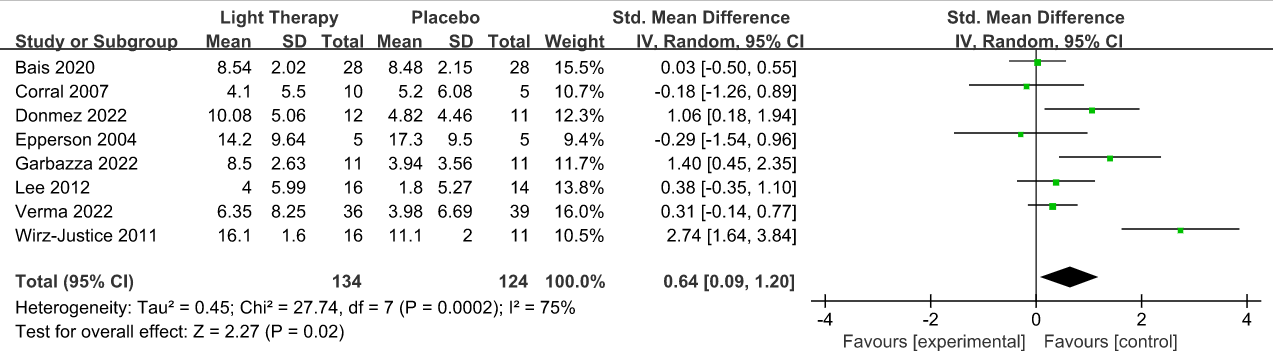
**

**S4. The overall quality of the evidence**

**GRADE table illustrating the respective certainty of evidence and clinical importance.**

| **Quality assessment** | | | | | | | **No of patients** | | **Effect** | | **Quality** | **Importance** |  |
| --- | --- | --- | --- | --- | --- | --- | --- | --- | --- | --- | --- | --- | --- |
|  |  |  |  |  |  |  |  |  |  |  |  |  |  |
| **No of studies** | **Design** | **Risk of bias** | **Inconsistency** | **Indirectness** | **Imprecision** | **Other considerations** | **Light therapy** | **Control** | **Relative (95% CI)** | **Absolute** |  |  |  |
| **Depression (Better indicated by lower values)** | | | | | | | | | | | | |  |
| 7 | randomised trials | no serious risk of bias | serious^1^ | very serious^2,3^ | very serious^4^ | none | 118 | 113 | - | SMD 0.34 higher (0.08 to 0.61 higher) | ⊕OOO VERY LOW | CRITICAL |  |
| **Sleep (Better indicated by lower values)** | | | | | | | | | | | | |  |
| 3 | randomised trials | no serious risk of bias | no serious risk of bias | very serious^2,3^ | very serious^4^ | none | 64 | 64 | - | SMD 0.64 higher (0.28 to 1 higher) | ⊕OOO VERY LOW | CRITICAL |  |

^1^ Moderate heterogeneity (I2=43%).
^2^ There are differences in the control methods used. Some studies used dim light and some used regular ward lighting.
^3^ There was variation in the interventions across the studies. Light therapy was used at different times and the intensity and time of light was not exactly the same.
^4^ All studies had less than 50 samples per group.
